# Imagine Jane and Identify John: Face Identity Aftereffects Induced by Imagined Faces

**DOI:** 10.1371/journal.pone.0002195

**Published:** 2008-05-21

**Authors:** Jae-Jin Ryu, Karen Borrmann, Avi Chaudhuri

**Affiliations:** Department of Psychology, McGill University, Montreal, Quebec, Canada; University of Granada, Spain

## Abstract

It is not known whether prolonged exposure to perceived and imagined complex visual images produces similar shifts in subsequent perception through selective adaptation. This question is important because a positive finding would suggest that perception and imagery of visual stimuli are mediated by shared neural networks. In this study, we used a selective adaptation procedure designed to induce high-level *face-identity aftereffects*—a phenomenon in which extended exposure to a particular face facilitates recognition of subsequent faces with opposite features while impairing recognition of all other faces. We report here that adaptation to either real or imagined faces produces a similar shift in perception and that identity boundaries represented in real and imagined faces are equivalent. Together, our results show that identity information contained in imagined and real faces produce similar behavioral outcomes. Our findings of high-level visual aftereffects induced by imagined stimuli can be taken as evidence for the involvement of shared neural networks that mediate perception and imagery of complex visual stimuli.

## Introduction

An encounter with a familiar person's name often generates the image of that person in our mind. The process by which an image is created without actual retinal input is referred to as visual imagery. Although there are reports of patients with deficits of perception but intact imagery [1], [2], multiple lines of evidence from behavioral [3], [4] and neuroimaging [5]–[7] studies suggest that the properties and neural substrates of imagery are similar to those of perception. The similarities in neural structures underlying imagery and perception are further corroborated by an electrophysiological study showing that single neurons in the human medial temporal lobe respond to both imagery and perception [8].

It is possible also to use psychophysical methods to study the neural mechanisms underlying perception and imagery. One approach is to use selective adaptation to directly probe the biological basis of cognitive function. In a selective adaptation experiment, an adapting stimulus is presented for an extended period of time, resulting in a temporary perceptual distortion, or aftereffect [9]. Aftereffects have been found with a wide range of visual stimuli, from simple lines [10], [11] to complex patterns such as faces [12], [13]. It is believed that sustained activity of neurons during adaptation causes a shift in their subsequent response level, leading to increased sensitivity to opposite (or complementary) stimulus attributes [14]. The method of selective adaptation is often referred to as the *psychologist's microelectrode* because it allows researchers to make inferences on the activity of neurons engaged in the processing of adapting stimuli [14].

The extant physiological evidence that similar neural structures are involved in perception and imagery leads to the hypothesis that perceived and imagined stimuli should produce similar behavioral results in a selective adaptation study. Despite this expectation, the results from several prior experiments have been inconsistent, reflecting the difficulty associated with using imagined visual stimuli in experimental settings [15]–[20]. Some of these studies only examined aftereffects induced by imagined stimuli with simple visual attributes, such as color and orientation, which are believed to activate early areas in the visual processing stream [21]. It is experimentally challenging to control various visual attributes such as precise hue orientation and size, of imagined stimuli [17], [18]. Furthermore, low-level visual aftereffects are sensitive to a variety of manipulations including changes in the size and position of adapting stimuli [9]. While problems that are inherent in the use of simple stimuli may partly explain some of the inconsistencies found in the extant literature, a previous study which did not find significant visual aftereffects induced by imagined complex stimuli such as faces [20] warrants further considerations of other factors such as participants' familiarity of the experimental tasks and stimuli.

In this study, we sought to directly probe the neural networks that underlie visual imagery and perception by inducing high-level, face-identity aftereffects (FIA) through selective adaptation. FIA occurs when adaptation to a particular face facilitates identification of subsequent faces with opposite features (anti-faces) while impairing identification of unrelated faces [12]. Unlike aftereffects induced by low-level stimuli, the biological mechanisms mediating FIA are invariant to changes in stimulus size, position, and orientation [12], [22].

For proper comparison of high-level aftereffects induced by imagined and perceived stimuli, the task requirements in both conditions should be identical. In order to ensure that the difficulty of imagining complex stimuli would not interfere with task performance, we used a fixed-order, within-subject design in which the aftereffect task with perceived stimuli preceded that with imagined stimuli. In addition, we added a discrimination task to ensure the identity information contained in perceived and imagined faces was equivalent. We hypothesized that if perception and imagery were indeed mediated by shared neural networks then adaptation to real and imagined faces would produce a similar bias in perception of subsequent faces.

## Results

Data were averaged over 8 participants whose baseline identification accuracy of test faces with 45% identity strength exceeded 75%. A logistic function 1/(1+exp(−(x−c)/a)) was fitted to the data, in which ‘a’ and ‘c’ are free parameters that determine the slope and midpoint of the psychometric function.

### Aftereffect tasks

At the end of the *imagined-stimulus* condition, all participants reported to be able to imagine a corresponding face during the adapting period. The *imagined-stimulus* condition was preceded by the *real-stimulus* condition for all participants. Therefore, their familiarity with the face stimuli was expected to be greater in the *imagined-stimulus* condition. The baseline tasks in both conditions involved identification of the morphed test faces without adaptation. The increased familiarity with the stimuli in the *imagined-stimulus* condition was reflected by the significantly increased performance on the baseline task compared to that of the *real-stimulus* condition [Greenhouse-Geisser correction for sphericity, *F* (1,7) = 8.313, *P*<0.05]. Due to the difference in the baseline performance between the *real-* and i*magined*-stimulus conditions, the differential effects of adaptation to matching (adapting face is the anti-face of the test face) and non-matching (adapting and test faces do not have opposite features) faces were analyzed for each stimulus condition.

**Figure 1:**
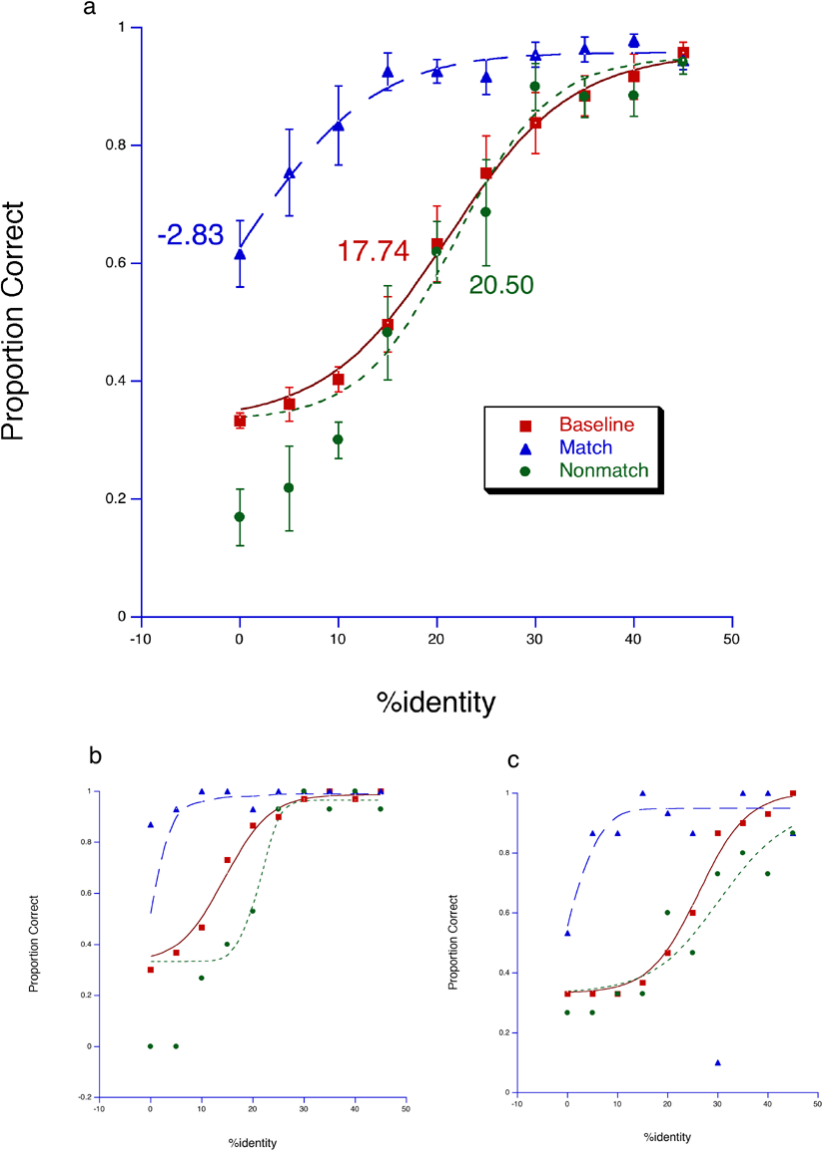
Sensitivity to face identity in *real-stimulus* conditions. The logistic function 1/(1+exp(−(x−c)/a)) was fitted to the data, in which ‘a’ and ‘c’ are free parameters that determine the midpoint and the slope of the psychometric function. The fraction of trials in which participants correctly identified the test face is plotted in relation to the identity percentage contained in test faces. Data from baseline with no adaptation (squares), adaptation to matching anti-face (triangles), and adaptation to non-matching anti-face (circles) are shown. The recognition threshold for each condition was taken to be the inflection point of the corresponding curve and shown accordingly. (a) Average of all participants. Standard errors are shown. (b and c) Individual data from two participants.

In the *real-stimulus* condition, there was a significant increase in identification performance after adaptation to “matching” faces compared with the baseline condition. In contrast, identification performance after adaptation to “non-matching” faces was diminished compared to baseline (Fig. 1). The fractions of trials in which participants correctly identified the test face in matching and non-matching trials were compared to the baseline performance. The respective differences in identification performance between the trial types (matching and non-matching) and baseline were subjected to a repeated-measure two-way analysis of variance (ANOVA) with trial type and identity strength as factors. The main effect of trial type [*F* (1,7) = 61.536, *P*<0.001], and interaction effect [*F* (2.832,19.824) = 11.364, *P*<0.001] were significant. The main effect of identity strength was marginally significant [*F* (4.467,31.266) = 3.651 *P*<0.05].

**Figure 2:**
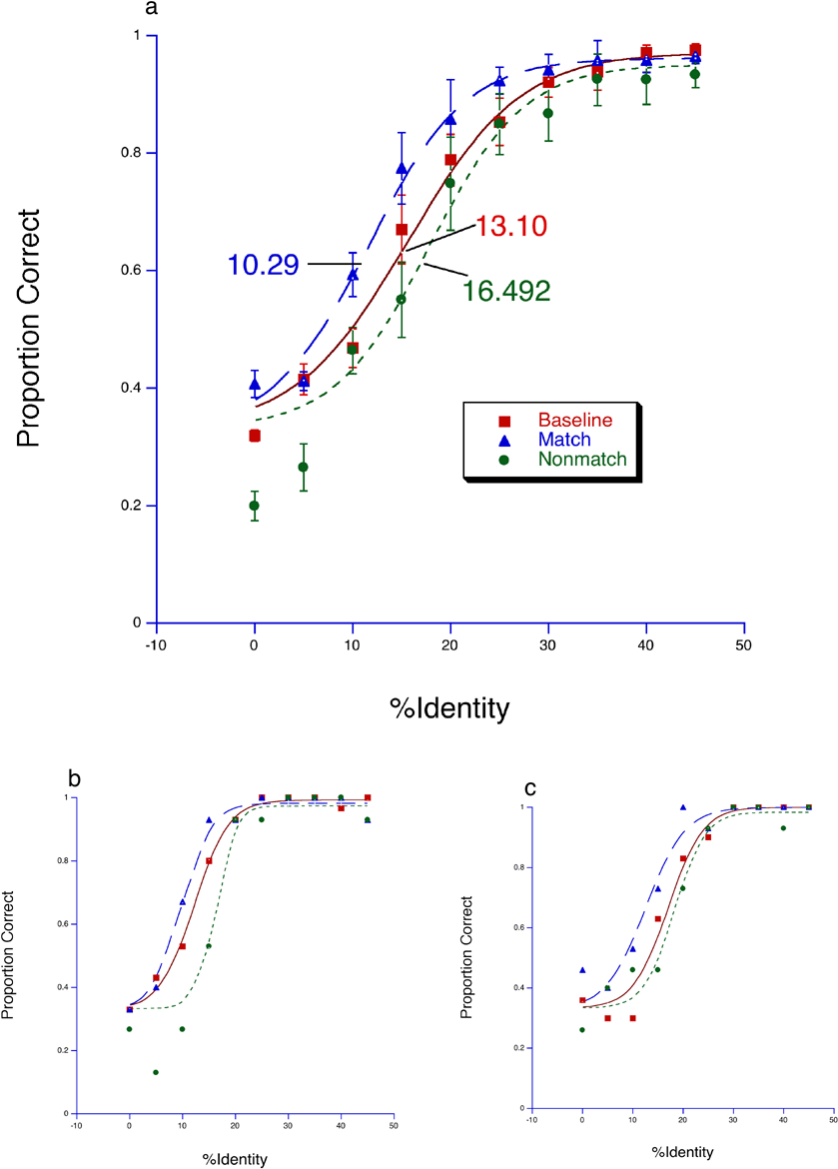
Results from *imagined-stimulus* conditions. (a) average of all participants, with corresponding threshold for each condition. Standard error of mean (SEM) are shown. (b & c) Data from two individual participants.

The *imagined-stimulus* condition produced a similar pattern of results to the *real-stimulus* condition (Fig. 2). An ANOVA revealed a significant main effect of trial type [*F* (1,7) = 28.613, *P*<0.01] and interaction effect [*F* (3.981,27.864) = 4.051, *P*<0.001]. The main effect of identity strength was not significant [*F* = (3.607, 25.251) = 1.397, *P*>0.05].

### Discrimination Task

**Figure 3:**
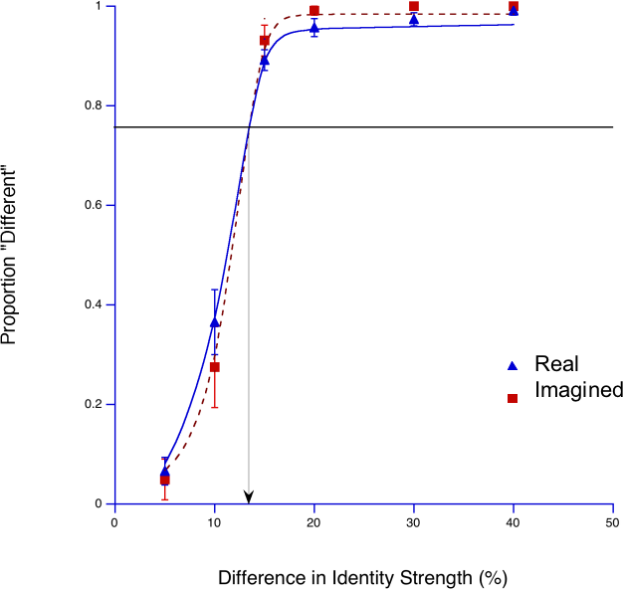
Difference thresholds for real (triangles) and imagined (squares) faces. The fraction of trials in which participants perceived the test face to be different from the previously learned face is plotted in relation to identity difference between the anti- and test faces. Difference thresholds (75% different responses) for the two psychometric functions for visual and imagined faces were identical.

The proportion of trials in which participants perceived the second face (Face 2) differently from the anti-face (Face 1) on the discrimination task was plotted against the difference in the identity strengths of the two faces (Fig. 3). The anti-face was either perceived or imagined. The data was fitted to a logistic function in order to extrapolate difference thresholds (75% different responses) for identifying visual and imagined faces. The difference thresholds were identical at 13%.

## Discussion

We have used selective adaptation methods designed to induce face-identity aftereffects (FIA) to test psychophysically whether face perception and imagery are processed by shared neuronal ensembles. We found that adaptation to physically presented matching anti-face images enhanced the recognition of test faces, whereas adaptation to anti-face images that were not matched to the test face resulted in reduced recognition performance compared to baseline. These effects can be seen from the corresponding shifts of the data points in Figure 1. Adaptation to imagined matching and non-matching anti-faces produced similar results—i.e., a significant increase in identification of test faces after adaptation to imagined ‘matching’ anti-faces and reduced identification performance relative to baseline after adaptation to non-matching imagined anti-faces (Fig. 2).

We also measured the respective difference thresholds for identity strength in a discrimination task to investigate this possibility and examine potential differences between the properties of real and mental images of learned faces. If imagined faces indeed had wider identity boundaries than real images of those same faces, this should be reflected in larger difference thresholds for imaged faces. However, we found that difference thresholds for real and imagined faces were the same, thus showing that the identity represented in the *imagined-stimulus* condition was similar to that contained in the *real-stimulus* condition.

Compared to the condition in which adapting stimuli were physically presented, the magnitude of shift from the corresponding baseline was much smaller when participants were asked to imagine the adapting stimuli (Figs. 1a & 2a). The apparent reduction of the aftereffect following adaptation to imagined stimuli could be explained by the difficulty in visualizing a complex image in a sustained, coherent manner. This may have lead to diminished activation of neuronal ensembles that otherwise show greater response to visual stimuli. The decreased activation of these neurons may have produced a smaller net adaptation leading to a smaller aftereffect.

We used a fixed-order, within-subject design to achieve participants' maximum familiarity with the experimental task and stimuli during adaptation to imagined stimuli. It is possible that the order in which the stimulus conditions were presented could have influenced participants' performance. Indeed, the increased familiarity with the face stimuli during the *imagined-stimulus* condition could have contributed to the reduced magnitude of aftereffect, as the baseline performance during the *imagined-stimulus* condition was significantly increased. However, despite the increased familiarity with the face stimuli, adaptation to non-matching imagined faces still reduced recognition performance, suggesting that the adaptation effect of non-matching imagined faces was comparable to that of real faces.

It is interesting to note that a similar study conducted by Moradi et al. [20] failed to report a significant high-level aftereffect induced by imagined faces. Due to the top-down nature of imagery, it is absolutely necessary to minimize possible cognitive and perceptual interferences during adaptation to imagined stimuli. In our task, the presentation of the name of an adapting face was brief, merely serving as cue for participants to imagine the corresponding face. Consequently, no visual stimulus was presented during adaptation. On the other hand, the name of an adapting face continued to be shown during adaptation in the study by Moradi et al. It is possible that continuous visual input during adaptation could have interfered with imagery, resulting in non-significant aftereffects.

This notion that neuronal responses are diminished during adaptation to an imagined stimulus is reinforced by a neuroimaging study that compared fusiform face area (FFA) and parahippocampal place area (PPA) activation during viewing or imagery of faces and places [7]. This study showed that both perception and imagery of faces selectively activated portions of FFA, whereas viewing and imagery of places produced greater activation in PPA. Interestingly, within a region responding more strongly to a given stimulus category, O'Craven and Kanwisher [7] also reported stronger levels of activation for real compared to imagined stimuli of that category. This finding is consistent with our finding of a larger magnitude of FIA for real as compared to imagined faces.

We have shown that adaptation to a visually presented anti-face and to an imagined anti-face produces similar perceptual aftereffects. The occurrence of such a high-level visual aftereffect from a purely mental image reveals that a close neural interaction exists between visual perception and imagery. The processing of complex visual stimuli such as faces is believed to be specialized in the later stages of the occipito-temporal visual processing stream [23], [24]. Accordingly, a recent neuroimaging study investigating the neural activity underlying FIA reported that areas in the anterior temporal lobe are involved in the mediation of the effect [25]. Given the similarity in the shift of perception following adaptation to imagined and real faces, these areas are likely to be involved in producing FIA induced by imagined faces.

Representations of a familiar face not only contain information about its visual attributes, but also semantic information regarding its identity. It appears that the two types of information are closely linked together. Support for this idea comes from a single-neuron study that reported neurons in the human medial temporal cortex that responded to both the presentation of a familiar face as well as the proper name associated with the face [26]. Since our aftereffect tasks involved perception and identification of familiar faces, some aspects memory processes may have contributed to our results. It is likely that both identity and visual information are activated during adaptation to perceived and imagined familiar faces.

Our results are consistent with neuroimaging studies that have shown selective activation of stimulus-specific brain regions in extrastriate cortex following exposure to both real and imagined stimuli. Similar activity following presentations of real and imagined stimuli have been found in face- [5], [7], object- [6] and place-selective [7] regions. However, our evidence supporting the commonality between neural structures underlying perception and imagery appears to be in conflict with previous reports of patients with dissociable deficits [1], [2], [27]. These patients showed severely impaired perception but more intact imagery of complex objects. It is possible, however, that intact imagery of these patients was mainly due to the retrieval of representations of objects acquired before the lesion. In that case, imagery tasks are likely to measure the patient's ability to remember, rather than to imagine what was just perceived. Although these studies provide interesting insight into the overall brain networks supporting perception and imagery, the dissociable deficits found in patients do not necessarily provide support for separate neural structures mediating these two experiences.

Our finding of equivalent identity boundaries for visual and mental images suggests that face imagery activates robust and accurate face representations that are similar to those produced by visual stimulation. We propose that the similarity in identity contained in imagined and real faces is produced by activation of shared neural networks that code for these representations.

## Materials and Methods

### Participants

Ten undergraduate students from McGill University participated in the study (2 males, mean age of 19.1 years). Participants were naïve to the purpose of the experiment. All had normal or corrected-to-normal vision. The study was reviewed and approved by an institutional ethics board for human psychophysical studies. Written consent was acquired from each participant prior to the experimental session. Data from two participants whose baseline identification accuracy of test faces with 45% identity strength task did not exceed 75% during the aftereffect tasks were removed from analysis.

### Apparatus and stimuli

All stimuli were presented on an LG flat-screen monitor with 1024×768 resolution and 85 Hz refresh rate. The stimuli were centrally presented on a uniform black background. The presentation sequence was programmed in MATLAB software using the Psychophysics Toolbox extensions [28]. A chinrest was used to stabilize head position at a distance of 57 cm from the monitor surface. All experiments were carried out in a dark testing room. The size of stimuli did not exceed 7×10.5 degrees.

Three face/anti-face pairs were taken from those used in the previous study reporting FIA [12]. According to the face space model, the anti-face of a face is located on the same identity axis but on the other side of the mean on the computationally derived, multi-dimensional face space [12], [29]. Therefore, the facial features of the face/anti-face pair are completely opposite to each other. The identity strength of each face was manipulated by adjusting its distance from the average face on the face space. Identity strength of face stimuli ranged from 0 (the average face) to 45%. Due to the computational processes through which anti-faces are generated, the maximal identity strength of an anti-face was 45%.

### Procedures

The experiment was divided into *real-* and *imagined*-*stimulus* conditions. Both conditions began with a training session in which participants were familiarized with the face stimuli. The identity strength of the faces was 45%. Each face appeared for five seconds on the monitor along with its fictional name (6 faces in total). The serial presentation of the faces was repeated seven times and the order of presentation was randomized. The participants' familiarity with the faces was probed with a verbal identification task in which each face was presented without its name. The presentation of the faces and identification task were repeated until 100% accuracy on the identification task was achieved.

In order to maximize the participants' familiarity with the anti-faces during the *imagined-stimulus* condition, all participants completed the *real-stimulus* condition before proceeding to the *imagined-stimulus* condition. In the *imagined-stimulus condition*, a discrimination task (described below) was added and was administered before the aftereffect task. In both *real-* and *imagined-stimulus* conditions, the baseline task, in which participants were required to identify test faces without adaptation, was completed after the aftereffect task.

### Aftereffect tasks

**Figure 4:**
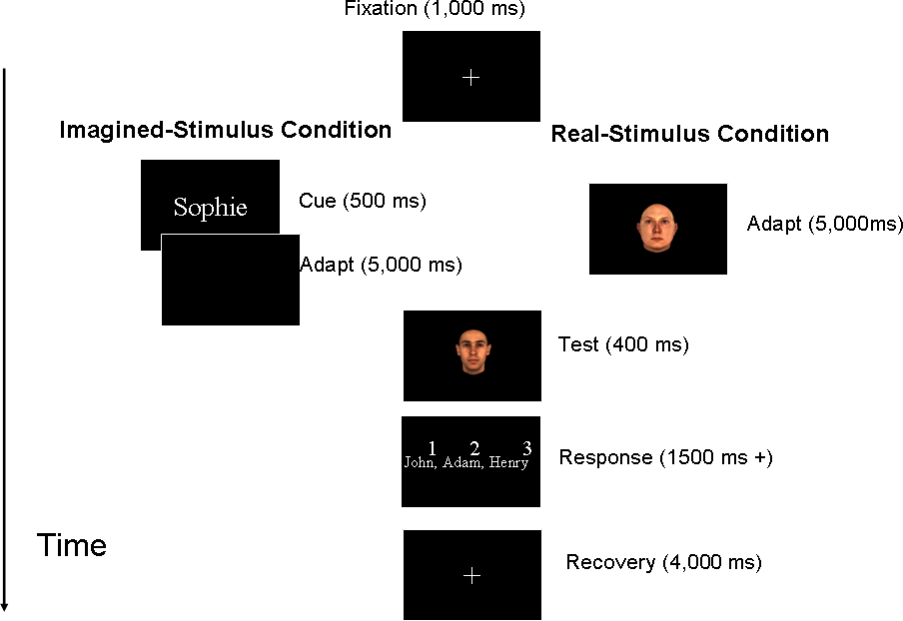
Trial sequence in the aftereffect tasks during the *real-* and *imagined-stimulus* conditions. A five-second presentation of the adapting stimulus was followed by a brief presentation of a test face. After adaptation, observers were asked to identity the test face among three shown faces.

In each trial of the aftereffect task, a five-second presentation of the adapting face was followed by a brief presentation (400 ms) of a morphed test face (Fig. 4). There was no ISI between the adapting and test images. The adapting face was one of the three anti-faces. The identity strength of a test face used in the aftereffect task ranged from 0 to 45%. The identity strength of anti-faces was 45%. In the *real-stimulus* condition, the adaptor was a true face image, whereas in the *imagined-stimulus* condition a name served as a cue prompting participants to vividly visualize the corresponding face with their eyes open. After adaptation, observers were asked to identify the test face among three shown face names and indicate their answers by pressing the appropriate button on the keyboard. Each adapting anti-face was shown 100 times during the aftereffect task, for the total of 300 trials in each condition.

The aftereffect tasks were composed of “matching” trials, in which the features of adapting and test stimuli were opposite to each other (face/anti-face pair) and “non-matching” trials, in which the two face stimuli were not perceptually related. An equal number (150) of matching and non-matching trials was randomly interleaved. To reduce a possible learning effect, we administered a baseline task after the respective aftereffect tasks. The baseline task required participants to identify test faces without adaptation. The test faces, as well as the number of trials presented during the baseline task, were identical to those shown during the aftereffect task.

### Discrimination task

**Figure 5:**
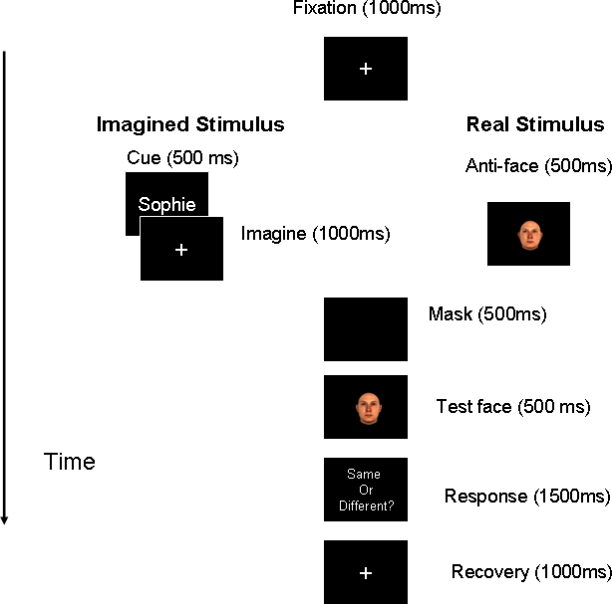
A trial in the discrimination tasks on real and imagined stimuli. Participants were asked to judge whether either the real or imagined anti-face and test face belonged to the same person. The identity strengths of the test faces were varied to be less than those of the anti-faces.

To assess possible differences in the degree of face identity contained in real and imagined stimuli, we sought to measure the difference threshold for identities in real and imagined faces in a discrimination task (Fig. 5). In this task, participants were instructed to compare either the real or the mental images of the anti-faces (Face 1) with subsequent real faces (Face 2). Participants indicated whether the two face stimuli (Faces 1 & 2) belong to the same person with a key press. Face 2 matched the identity of Face 1 (i.e., both faces can be found on the same identity trajectory on the face-space), but the identity strengths of Face 2 stimuli were less than those of the Face 1 stimuli: The identity strengths of Face 1 stimuli were 45% whereas the identity strengths of Face 2 stimuli varied from 5 to 40%. Each Face 2 stimulus appeared 5 times with each of the real and imagined Face 1 stimuli. Participants completed 180 trials in total.
